# Drivers of and Barriers to COVID-19 Vaccine Booster Dose Acceptance in Indonesia

**DOI:** 10.3390/vaccines10121981

**Published:** 2022-11-22

**Authors:** Harapan Harapan, Raisha Fathima, Hendrix Indra Kusuma, Samsul Anwar, Widhy Yudistira Nalapraya, Adityo Wibowo, Ketut Dewi Kumara Wati, Ayunda Medina, Anna Hanifa Defrita, Yesi Astri, Arie Prasetyowati, Nurfarahin Nurfarahin, Afriyani Khusna, Setya Oktariana, Sarifuddin Anwar, Milza Oka Yussar, Siti Khotimah, Bahagia Willibrordus Maria Nainggolan, Putri Rizki Amalia Badri, Raden Argarini, Wira Winardi, Rosaria Indah, Malik Sallam, Yogambigai Rajamoorthy, Abram L. Wagner, Mudatsir Mudatsir

**Affiliations:** 1 Medical Research Unit, School of Medicine, Universitas Syiah Kuala, Banda Aceh 23111, Indonesia; 2 Tropical Disease Centre, School of Medicine, Universitas Syiah Kuala, Banda Aceh 23111, Indonesia; 3 Department of Microbiology, School of Medicine, Universitas Syiah Kuala, Banda Aceh 23111, Indonesia; 4 Tsunami and Disaster Mitigation Research Center (TDMRC), Universitas Syiah Kuala, Banda Aceh 23111, Indonesia; 5 Department of Biology, Faculty of Mathematics and Natural Sciences, Universitas Syiah Kuala, Banda Aceh 23111, Indonesia; 6 Biology Education Department, Faculty of Tarbiyah and Teacher Training, Universitas Islam Negeri Ar-Raniry, Banda Aceh 23111, Indonesia; 7 Department of Statistics, Faculty of Mathematics and Natural Sciences, Universitas Syiah Kuala, Banda Aceh 23111, Indonesia; 8 Department of Pulmonology and Respiratory Medicine, Faculty of Medicine, Universitas Islam Bandung, Bandung 40116, Indonesia; 9 Department of Pulmonology and Respiratory Medicine, Faculty of Medicine, Universitas Lampung, Bandar Lampung 35145, Indonesia; 10 Department of Child Health, Faculty of Medicine, Universitas Udayana, Denpasar 80234, Indonesia; 11 Faculty of Medicine, Universitas Jambi, Jambi 36373, Indonesia; 12 Neurology Department, Faculty of Medicine, Universitas Muhammadiyah Palembang, Palembang 30263, Indonesia; 13 Mungkid Community Health Center, Magelang 56512, Indonesia; 14 Department of Pulmonology and Respiratory Medicine, Faculty of Medicine, Tadulako University, Palu 94148, Indonesia; 15 Faculty of Public Health, University Muhammadiyah Aceh, Banda Aceh 23245, Indonesia; 16 Biochemistry Laboratory, Medical Faculty of Mulawarman University, Samarinda 75119, Indonesia; 17 Undergraduate Program in Medicine, Faculty of Medicine, Universitas Sumatera Utara, Medan 20155, Indonesia; 18 Public Health Department, Faculty of Medicine, Universitas Muhammadiyah Palembang, Palembang 30263, Indonesia; 19 Department of Medical Physiology and Biochemistry, Universitas Airlangga, Surabaya 60132, Indonesia; 20 Department of Pulmonology and Respiratory Medicine, School of Medicine, Universitas Syiah Kuala, Banda Aceh 23111, Indonesia; 21 Medical Education Unit, School of Medicine, Universitas Syiah Kuala, Banda Aceh 23111, Indonesia; 22 Department of Pathology, Microbiology and Forensic Medicine, School of Medicine, The University of Jordan, Amman 11942, Jordan; 23 Department of Clinical Laboratories and Forensic Medicine, Jordan University Hospital, Amman 11942, Jordan; 24 Department of Translational Medicine, Faculty of Medicine, Lund University, 22184 Malmö, Sweden; 25 Department of Economics, Faculty of Accountancy and Management, Universiti Tunku Abdul Rahman, Kuala Lumpur 43200, Malaysia; 26 Department of Epidemiology, School of Public Health, University of Michigan, Ann Arbor, MI 48109, USA

**Keywords:** booster dose, vaccine acceptance, vaccine reluctance, COVID-19, vaccine resistance, vaccine rejection, vaccine hesitancy

## Abstract

Obtaining a booster dose of coronavirus disease 2019 (COVID-19) vaccine is required to maintain the protective level of neutralizing antibodies and therefore herd immunity in the community, and the success of booster dose programs depends on public acceptance. The aim of this study was to determine the acceptance of a booster dose of COVID-19 vaccine and its drivers and barriers in Indonesia. A cross-sectional survey was conducted in the provinces of Indonesia between 1 and 15 August 2022. Individuals who completed the primary series of the COVID-19 vaccine were asked about their acceptance of a booster dose. Those who refused the booster dose were questioned about their reasons. A logistic regression was used to determine the determinants associated with rejection of a booster dose of COVID-19 vaccine. A total of 2935 respondents were included in the final analysis. With no information on the efficacy and safety of the COVID-19 vaccine, 95% of respondents agreed to receive a booster dose if it were provided for free by the government. This acceptance was reduced to only 50.3% if the vaccine had a 75% efficacy with a 20% chance of side effects. The adjusted logistic regression analysis indicated that there were eight factors associated with the rejection of the booster dose: age, marital status, religion, occupation, type of the first two vaccines received, knowledge regarding the importance of the booster dose, belief that natural immunity is sufficient to prevent COVID-19 and disbelief in the effectiveness of the booster dose. In conclusion, the hesitancy toward booster doses in Indonesia is influenced by some intrinsic factors such as lack of knowledge on the benefits of the booster dose, worries regarding the unexpected side effects and concerns about the halal status of the provided vaccines and extrinsic determinants such as the effectiveness and safety of the vaccine. These findings suggest the need for more campaigns and promotions regarding the booster dose benefits to increase its acceptance.

## 1. Introduction

Coronavirus disease 2019 (COVID-19), caused by severe acute respiratory syndrome coronavirus 2 (SARS-CoV-2), is still a major public health problem with a significant impact globally, including in countries in Southeast Asia such as Indonesia [1]. The World Health Organization (WHO) data indicated that COVID-19 cases are still increasing in the region, including in Indonesia. Between 4 and 10 July 2022, there was an increase of 117% in cases and 212% in deaths in Indonesia [1]. According to the Indonesian National Task Force for COVID-19, the overall number of confirmed COVID-19 cases in the country was more than 6.2 million, with the total death toll reaching 157 thousand [2]. Interestingly, the data showed that an uptake across the subsequent vaccine doses has declined gradually; there is a 1.1-fold decrease from the first to the second dose and a 3-fold decrease from the second to the booster dose [2]. The acceptance of a booster dose of the COVID-19 vaccine is clearly a national challenge. Only 27.4% of people out of the national vaccine coverage target (208,265,720 people) have received a booster dose [2,3]. 

The evidence shows waning protection from COVID-19 vaccines against infection over time due to declining immunity and the emergence of the new variants [4,5] raising concerns that the primary series of COVID-19 vaccinations will not be adequate to maintain a long-term protective effect against SARS-CoV-2 infection. Booster doses are therefore needed. The effectiveness of the booster dose program will depend on public acceptance [6,7]. 

As Indonesia is still struggling to distribute booster doses to an adequate number of people, understanding the basis to vaccine acceptability is critical for developing strategies to immediately form community health resilience [8]. Previous studies in various countries have highlighted several modifiable and unmodifiable factors associated with the acceptance of vaccines: knowledge, gender, doubt about vaccine information, experiences and the awareness of vaccine importance [9,10,11,12,13,14,15]. 

In addition, some of the uncertainties related to the COVID-19 vaccine’s safety also contribute to vaccine acceptance [13]. Side effects of the COVID-19 vaccine are relatively high [16,17], and a systematic review on published COVID-19 vaccine trials found the side effects could be local (such as pain, swelling and redness at the site of injection) or systemic reactions, such as fever, myalgia, fatigue or headache, decreased hemoglobin or increased bilirubin or liver enzymes [18]. Severe side effects of COVID-19 vaccines also have been reported, such as thrombosis [19,20,21,22], myocarditis [23,24,25], acute disseminated encephalomyelitis [26] and encephalitis [27,28]. Possible deaths associated with the COVID-19 vaccination have also been reported [19,20,21,22,23,24,25,26,29,30]. A systematic review assessing the causal relationship of death after the COVID-19 vaccination found 55 deaths after receiving the COVID-19 vaccination as of November 2021, of which 14 were very probable/demonstrated, while others were not specified (*n* = 8), considered possible (*n* = 15) and probable (*n* = 1) [31]. The current studies also found increased risks of myocarditis after COVID-19 vaccination [25,30]. 

A study about the acceptance of the COVID-19 vaccine booster in two provinces of Indonesia showed that the socioeconomic determinants, health beliefs, social media and trust in government information were associated with booster dose acceptance [32]. An update to these findings, which includes other provinces that had low vaccine coverage, will contribute to a better understanding of the recent condition underlying low booster dose coverage in the country and may also be useful for other countries in Southeast Asia. The aim of our study was to determine the acceptance of a booster dose of COVID-19 vaccine and its drivers and barriers within the five main island groups of the Indonesian archipelago.

## 2. Materials and Methods

### 2.1. Study Design

A cross-sectional survey was conducted in Indonesia covering five main islands in the country. Individuals who were Indonesian citizens, over 18 years old and had completed the primary series of COVID-19 vaccination were eligible to participate in this study. The respondents were asked about their acceptance of a booster dose. Those who were hesitant were asked about their reasons and barriers. The Ethical Committee of the School of Medicine, Universitas Syiah Kuala, examined and approved the study protocol (No. 008/EA/FK/2022 and registration number 1171012P). All respondents who participated in the study provided their informed consent. 

### 2.2. Study Variables and Study Instrument

The dependent variable of the study was the acceptance of a booster dose of COVID-19 vaccine. To assess the acceptance, the respondents were asked whether they would accept a booster dose of COVID-19 vaccine if it were provided freely by the government without information about the efficacy or safety of the booster dose. Subsequently, in order to assess the role of different vaccine profiles, respondents were then asked about their acceptance of a booster dose of the vaccine with different efficacies (95%, 75% or 50%) and different chances of side effects such as fever or local pain (5% or 20%). 

The plausible explanatory variables of booster dose acceptance in this study ranged from sociodemographic data (gender, age, marital status, last attained education, religion, type of job and monthly salary); knowledge and belief about the safety and efficacy of the COVID-19 vaccine; perceived risk of COVID-19 infection; previous COVID-19 infection; experiences surrounding previous COVID-19 vaccinations and knowledge about the vaccine benefits. 

The questionnaire consisted of several different sets of questions. In the first section, respondents were asked demographic information (age, gender, province of residence, marital status, last attained education, religion, job and monthly salary). The second section asked about knowledge of a booster dose of the COVID-19 vaccine where the respondents’ knowledge regarding the booster dose effects was asked in four questions that could be responded with “yes”, “no” or “not sure”: (1) whether a booster dose can give better immunity, (2) whether a booster dose can stimulate the production of antibodies for protection against COVID-19, (3) whether a booster dose can lower the hospitalization rate of COVID-19 infection and (4) whether a booster dose can protect unvaccinated people. The accurate answer to an item was scored as one, whereas an incorrect/uncertain response was scored as zero. The third section was about the awareness of the importance of a booster dose of the COVID-19 vaccine. 

The acceptance for a booster dose of the COVID-19 vaccine is assessed in Section four, including the booster doses’ country of origin. Section five evaluates the respondents’ perceptions of booster doses with four questions: (1) whether the respondent worried about the side effects or allergic reactions towards a booster dose, (2) whether there is a belief that the booster dose is crucial, (3) whether a booster dose is useful in protecting people from COVID-19 and (4) whether a booster dose is safe. The possible responses ranged from “strongly agree” to “strongly disagree”. For each “strongly agree” and “agree” response, respondents were given a score of one, while each “neither agree or disagree”, “disagree” and “strongly disagree” response received a score of zero.

The next three sections asked about the perceived severity of COVID-19, the perceived benefit of the booster dose and the perceived barriers to getting the booster dose in Indonesia. Perceived severity was assessed using two questions: (1) COVID-19 may lead to a severe condition and (2) fears of unexpected side effects of a booster dose of COVID-19 vaccine in the future. Perceived barriers on getting the booster dose were assessed using two questions: (1) whether the respondents doubted the halal status about the booster dose and (2) whether it takes a lot of time and effort to get the booster dose. 

In addition, in the last section, to further explore the acceptance and hesitancy of a booster dose of the COVID-19 vaccine, the respondents were asked about their motivations to be vaccinated and the factors that influenced their decision to accept the booster dose. One respondent could choose more than one motivation or factor. 

### 2.3. Data Collection Procedure

The data collection was conducted between 1 and 15 August 2022 on the Survey Monkey platform. It is an online survey software using the web interface that allows the authors to use multiple pages and logics during the survey (www.surveymonkey.com). To cover all the main island groups in Indonesia, 31 local enumerators were recruited who were responsible for data collection. The electronic links were distributed to community members through the local enumerators’ social networks, including WhatsApp, Telegram, Messenger, Line, Facebook, Instagram and Twitter. Before completing the questionnaire voluntarily, each potential respondent provided informed consent by clicking the “Agree” button. The next page of the survey was opened automatically if the respondent agreed to participate. When the respondent clicked the “Agree” button, any responses provided by the respondents were recorded and collected automatically, even if the survey was not completed. To ensure anonymity and confidentiality, no identifiable details of personal information were collected. There was no compensation promised or provided to the respondents.

### 2.4. Data Analysis

All analyses were conducted using SPSS version 20 (IBM SPSS Inc., Chicago, IL, USA). For continuous variables, descriptive statistics were reported in means and standard deviations (mean ± SD), whereas categorical variables were summarized using frequencies and percentages. In this exploratory analysis of reasons behind the booster dose vaccination acceptance, a two-step logistic regression procedure was implemented. First, the crude odds ratio (OR), along with 95% confidence interval (CI), were computed for each independent variable. All drivers or barriers that were significant in this unadjusted logistic regression analysis (defined with *p* < 0.05) were included in the adjusted analysis, with the output being an adjusted odds ratio (aOR). Statistical significance was defined as *p* < 0.05.

## 3. Results

### 3.1. Sociodemographic Characteristics of Respondents

We received 3695 responses during the study, and 760 (20.6%) were excluded due to incomplete responses. A total of 2935 (79.4%) respondents who responded to all of the questions were included in the analysis (Table 1). Out of the total respondents, 68% (1993/2935) were female, almost half of the respondents (47.9%) were between 21 and 30 years old and 45.2% were married. More than half of the respondents (67%; 1973/2935) graduated from a university, and 11.5% were postgraduate and doctoral graduates. About 52% of them were employed for wages, 55.8% (1638/2935) earned <3 million (equal to 200.9 USD) per month and 81% of the survey respondents were Muslim. The complete characteristics of the respondents are presented in Table 1.

### 3.2. Characteristics of Experience, Knowledge, Perception, Perceived Severity, Perceived Benefit and Perceived Barriers

A total of 740/2935 respondents (25%) reported having a family member who was seriously ill or who died due to COVID-19, and 47.3% had been infected with COVID-19 themselves (Table 2). Relatively few (16.7%) had been vaccinated against influenza in the past 5 years. More than 80% had received first and second doses of the Sinovac vaccine against COVID-19, and nearly 30% reported being infected with COVID-19 even after being vaccinated (Table 2). 

More than 80% of the respondents believed that a booster dose could improve immunity, stimulate antibody production, and reduce the number of COVID-19 treatments (Table 2). Almost 70% of the respondents believed that the vaccination could help to protect people who could not be vaccinated (people with a comorbidity or the elderly). However, 24.5% of the respondents were doubtful that pharmaceutical companies had developed a safe and effective booster dose of the COVID-19 vaccine, 34.4% of them believed they had strong immunity and 19.6% believed COVID-19 was not harmful. About 36% were uncertain that the booster dose of the vaccine was effective against COVID-19, and 87.3% feared possible side effects in the future. In addition, 71.9% of Muslim respondents were concerned about the halal status of the vaccine they would receive (Table 2).

In relation to vaccine decisions, 57.9% reported that their decision to get vaccinated with a booster dose was influenced by a person or family they live with. More than half of the respondents (57.5%) also admitted that their decision was influenced by their colleagues at their workplace during the pandemic. In terms of perceived benefits, 79.0% and 81.4% of the respondents believed that booster doses of the vaccines were useful for protecting themselves and others from COVID-19 infection, respectively. Although a majority of respondents (81.8%) believed that a booster dose reduced the risk of contracting and infecting others with COVID-19, 20.5% of them doubted that the benefits of the booster dose outweighed the risks.

### 3.3. Acceptance of the Booster Dose of COVID-19 Vaccine

Without revealing the efficacy and safety of the COVID-19 vaccine, 93.9% of the respondents would accept the booster dose if it were freely provided by the Indonesian government, and only 6.1% of the respondents would reject the booster dose (Table 3). If the booster dose of the COVID-19 vaccine had a 75% effectiveness with a 5% chance of side effects, only 84% (2461/2935) of respondents were willing to be vaccinated. This proportion decreased to 69.2% if the chance of side effects was 20%, even if the vaccine had a 95% effectiveness. The acceptance of a booster dose was only 50.3% for a vaccine 75% effective in preventing SARS-CoV-2 infection and had a 20% chance of potential side effects (Table 3).

### 3.4. Factors Associated with Booster Dose Acceptance 

The initial logistic regression analysis showed that demographic factors such as age, marital status, religion and occupation were associated with booster dose vaccine acceptance (Table 4). Besides demographic factors, respondents who have been infected with COVID-19 were 1.5 times more likely to accept the vaccine compared to those who had not (Table 4). Compared to respondents who disagreed that COVID-19 has greatly affected their source income, the respondents who agreed with the terms were twice more likely to accept the vaccination. Those who agree that the COVID-19 pandemic has greatly affected their social life have five times higher odds of acceptance compared to those who disagreed. Respondents who believed that pharmaceutical companies have developed a safe and effective vaccine and that the booster dose was important to protect the public had a 26–38 greater odds ratio of accepting the vaccine compared to those who did not. If the government provided the vaccine for free, the respondents were 3.5 times more likely to accept compared to if the vaccine was not provided for free. Agreeing that the decision to get vaccinated was greatly influenced by the family or the person who lived with them at home and not sure if vaccination is effective against COVID-19 was associated with vaccine acceptance. Compared to respondents who did not agree, those who agreed that the booster dose for the COVID-19 vaccine will have good effectiveness will be useful in protecting them from COVID-19; the benefits of the vaccine outweighed the risk, and if they get vaccinated, the risk of contracting COVID-19 or infecting others will be reduced and have a 34–98 greater odds ratio of accepting the vaccine. These factors could be considered as vaccine acceptance drivers, because they increased the acceptance rate.

Respondents who were vaccinated by a Pfizer vaccine had a lower acceptance odds ratio compared to those who received Sinovac for the 1st and 2nd dose (OR: 0.24; 95%CI: 0.13–0.43 and OR: 0.30; 95%CI: 0.18–0.52, respectively). Not knowing that they have been reinfected with COVID-19 after vaccination, belief that a booster dose can provide better immunity than just a second dose and that a booster dose can stimulate antibody production, lower hospitalization, and protect other people who were not vaccinated were also associated with booster dose acceptance (Table 4). Participant perception of potential side effects that may occur when vaccinated with a booster dose vaccination and the belief that vaccination is important, that vaccination should be a condition for travel and that the vaccine is useful to protect people from COVID-19 were all associated with vaccine acceptance. 

The factors associated with reduced acceptance included the belief that serious complications will arise after getting the COVID-19 booster vaccine and worry about the unexpected side effect of the COVID-19 booster dose vaccine in the future. Respondents who agree that they were worried about the halal status of the vaccine and that getting a booster dose vaccination takes a lot of time and effort were less likely to receive the vaccine (OR: 0.20; 95%CI: 0.12–0.34 and OR: 0.57; 95%CI: 0.37–0.89). These factors were classified as inhibitors, because they reduced the rate of acceptance. 

All factors that were significant in the unadjusted model were included in the final logistic regression model. In the adjusted logistic regression model, only some factors were significantly associated with the acceptance of a booster dose of the COVID-19 vaccine. These included age, marital status, religion, occupation, type of vaccine received in the first and second dose by the respondents, belief in the efficacy of the booster dose in protecting the public from COVID-19, belief in the role of natural immunity and belief in the effectiveness of the vaccination against COVID-19 (Table 4). 

Respondents between 18 and 30 years old had two-to-three times higher acceptance for the booster dose compared to respondents 31–40 years old (Table 4). Participants who were employed for wages had twice the odds of acceptance compared with those who worked as homemakers (aOR: 2.57; 95%CI: 1.07–6.18; *p* = 0.035). The participants who received a Sinovac vaccine for the first dose had a higher acceptance compared to those who received Sinopharm, while those who received AstraZeneca during the second dose had almost an eight times higher odds ratio of acceptance compared to those who received the Sinovac vaccine. Respondents who did not know if they had ever been infected with COVID-19 after getting vaccinated had lower odds of accepting a booster dose compared to those who knew that they had been infected (aOR: 0.42; 95%CI: 0.21–0.86; *p* = 0.018). Compared to the respondents who did not believe that the booster dose is important to protect the public from COVID-19, those who believed were three times more likely to accept the booster dose. 

Those who agreed that the booster dose for the COVID-19 vaccine is very important were almost six times more likely to accept the booster dose compared to those that disagreed (aOR: 5.54; 95%CI: 1.90–16.15; *p* = 0.002). The participants who believed that natural immunity is sufficient to protect from COVID-19 had a lower acceptance rate compared to those who did not have this belief (aOR: 0.43; 95%CI: 0.23–0.83; *p* = 0.011). Respondents who did not believe or were unsure that the vaccine is effective against COVID-19 had lower odds of booster dose acceptance compared to those who believed (aOR: 0.42; 95%CI: 0.21–0.83; *p* = 0.012 and aOR: 0.46; 95%CI: 0.24–0.87; *p* = 0.017, respectively).

### 3.5. Motivations, Factors That Influence and Source of Vaccine Associated to Booster Dose Acceptance

**Figure 1:**
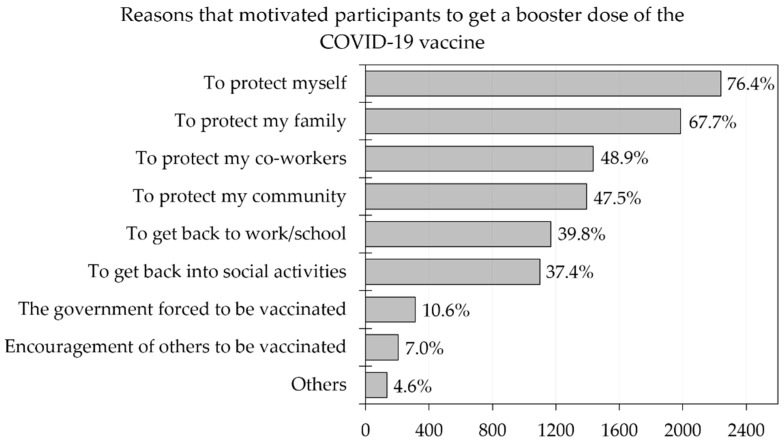
Reasons that motivated participants to get a booster dose of the COVID-19 vaccine (*n* = 2935).

To further explore the acceptance and rejection of a booster dose of the COVID-19 vaccine, the motivations to be vaccinated and factors that influenced their decision to accept the booster dose were determined. Among all respondents, the highest motivator was to protect themselves (76.4%), followed by protecting their family (67.7%) and protecting their co-workers (48.8%). There were some respondents (10.6%) who stated that government pressure was one of their reasons for accepting a booster dose (Figure 1). 

**Figure 2:**
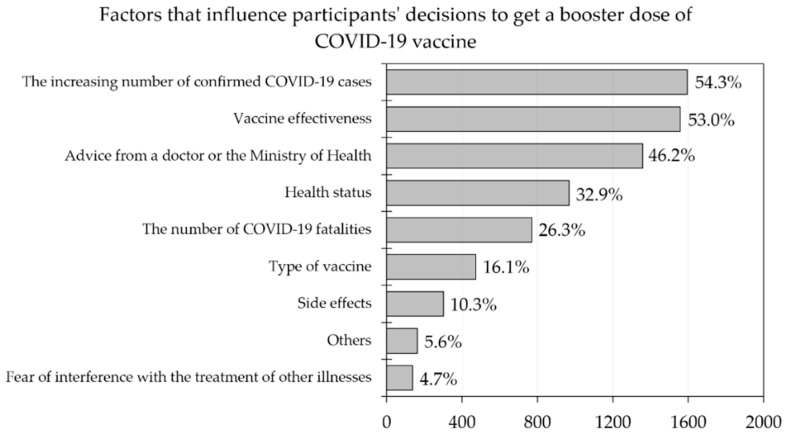
Factors that influenced the participants’ decisions to get a booster dose of COVID-19 vaccine (*n* = 2935).

In the decision process to accept a booster dose, the respondents stated that some reasons included increasing the number of confirmed COVID-19 cases in their area (54.3%), vaccine effectiveness (53.0%), advice given by doctors or the Ministry of Health (46.2%), the participants’ health status (32.9%) and fatalities caused by COVID-19 (26.3%). The type of vaccine, side effects and fear of interference with the treatment of other illnesses were less important reasons, being listed by 16.1%, 10.3% and 4.7% of the respondents, respectively (Figure 2). 

**Figure 3:**
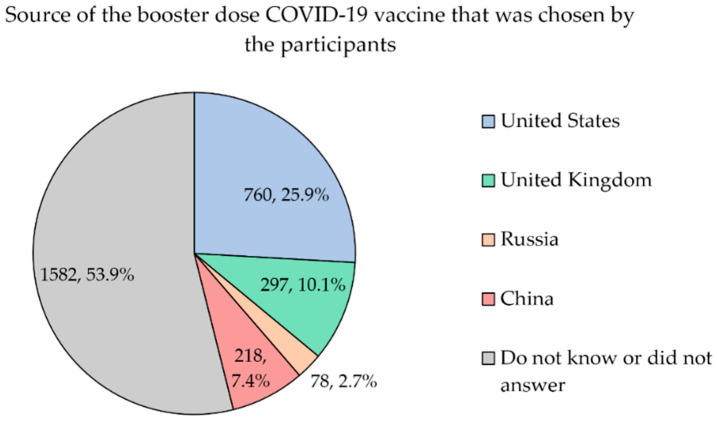
Preferred source of the COVID-19 booster dose chosen by the participants (*n* = 2935).

In terms of vaccine type, participants also acknowledged the preferred sources of the booster dose, such as the United States (25.9%), United Kingdom (10.1%), China (7.4%) and Russia (2.7%). More than half of the participants (53.9%) did not know/did not answer for their country of choice for a booster dose (Figure 3).

## 4. Discussion

This survey was conducted across Indonesia on a backdrop of continued outbreaks of COVID-19 and a need for understanding why individuals were vaccine-hesitant. In this study, 93% of the respondents would accept a booster dose of the COVID-19 vaccine if it were provided by the government for free. This acceptance rate is above the target of the Indonesian Ministry of Health, which stated that 70% vaccine coverage is adequate to achieve herd immunity [33]. This rate is also higher compared to the acceptance rate in Japan (4832/6172; 78.3%) [34], China (6321/8229; 76.8% accepted) [35], Poland (330/443; 74.5%) [36] and India (384/687; 52.1) [37]. However, this present study is limited to those who have already completed the primary series (an estimated 60.8% of the population). We also found that the booster dose acceptance was highly dependent on the vaccine profile. For example, even with a 95% efficacy, if the chance of any side effects was 20%, the acceptance rate was only 69.2%, which is below the national vaccine coverage target. This finding indicates that concerns regarding side effects may inhibit the community in receiving a booster shot. This is understandable, since the chance of mild side effects after the COVID-19 vaccination is relatively high [16,17,18], and some severe side effects [19,20,21,22,23,24,25,26,27,28], including unexpected cardiac arrest [30] and sudden deaths [38], have been reported. Our data also suggest that information about the vaccine effectiveness would increase acceptance. Therefore, the more knowledge that the community has about the vaccine effectiveness could increase society’s decision to get the booster shots. This is in line with our previous findings, which suggested that public education on the benefits of vaccines needs to be improved in order to increase vaccine uptake [39]. Another study also found people who believe that the COVID-19 vaccine is effective were 7.95 times more likely to receive a third or booster dose compared to those who do not [40]. Therefore, information on the effectiveness of the vaccine should be well-communicated through simple and effective techniques to community members so that they are well-informed. 

Our study found that the specific vaccine received by participants in the past led to differing levels of acceptance of a booster. Sinovac was the type of vaccine that most respondents received as the first dose (85.1%), followed by AstraZeneca (7.6%), Moderna (3.1%) and Pfizer (2.6%). Our study indicated that recipients of the Sinopharm or AstraZeneca vaccine were significantly more hesitant to accept a booster dose compared to those with a Sinovac vaccine. This finding might be associated with the safety or effectiveness profile of the previously received vaccine. A study indicated that six months after two doses of Sinovac, there were low antibody concentrations [41]; therefore, a booster dose was needed. Moreover, a heterologous booster dose resulted in more robust immune responses than a homologous booster dose [41,42]. Knowledge of this fact might encourage an individual to get the booster dose. In addition, a study clearly found that that Sinopharm and Sinovac vaccines were associated with more frequent side effects, such as pain at the injection site, headache, fatigue and fever, compared to Sinovac: 62.5% and 35.7% vs. 28.5% [43]. This indicates that experienced side effects from previous vaccines could impact what the individual thinks about the safety of subsequent doses, and, accordingly, their acceptance of a subsequent dose.

Two inhibitors toward booster dose acceptance were identified: belief that natural immunity is sufficient in preventing COVID-19 and belief that the vaccination is not effective against COVID-19 (Table 4). A previous study also found vaccination refusal due to belief in the sufficiency or primacy natural immunity, indicating that this belief is not a new phenomenon [44]. Studies have shown that some parents believe that natural immunity achieved from disease is superior to vaccination-induced immunity [45,46]. They believe that natural immunity is more beneficial in the long term compared to vaccination results and that the vaccination has other drawbacks, such as the introduction of undesired chemicals into their bodies [47]. These beliefs could also be related to the side effects of the vaccine that they were unaware of, given that the vaccine offered is new, and its effectiveness is unknown [47]. This also accords with beliefs about the effectiveness of the vaccine against COVID-19. Clearly, the public wishes to be immunized with a vaccine that is truly effective in protecting against the disease, and if they do not think that the vaccine does this, they could believe that a booster dose is unnecessary. In addition, there may be certain circumstances that lead to a belief that vaccination, even a booster dose, is ineffective, such as reinfection after getting the booster dose and a quick process of vaccine development. A study in Egypt showed that there was a concern over the vaccine’s ineffectiveness (93.2%) [48]. The study also found that the most confirmed hurdle to COVID-19 vaccination acceptance was a lack of information about the vaccine itself (72.76%) [48]. This ineffectiveness may also be sensed, because the pre-pandemic mobility people regain nowadays after doses of the COVID-19 vaccine and reinfection rates are rising again [49]. 

The perceived halal status of the vaccine doses significantly contributes to the respondents’ confidence in accepting a booster dose. A previous study also found that those who refused the vaccine believed that it was not halal. Several respondents were under the impression that vaccines might contain non-halal ingredients such as porcine and fetal materials [50]. We note that Indonesia’s supreme religious body, the Indonesia Ulema Council, has assured the halal status of Sinovac and determined that several vaccines were mubah (permissible to use in an emergency), such as AstraZeneca, Pfizer and Moderna [32,51]. 

Motivations to receive the booster dose of the COVID-19 vaccine may vary. Based on a study conducted in Spain, the concern of transmitting COVID-19 to the family (49.52%) and concern of self-infection (39.45%) were the most common reasons for vaccination, followed by socializing (31.0%) and travel (30.56%) [52]. Another study in Jordan also highlighted the similar findings regarding the acceptance of an annual dose of the COVID-19 vaccine, such as the potential of the additional dose in lowering the risk of contracting COVID-19 disease (62.2%), protecting family members from the virus and consequences, protecting their job (34.2%) and reducing the cost of hospital care (28.6%) [53]. These findings were similar to what we found—that self-protection, protecting family members, co-workers and the surrounding community motivate the respondents to receive the booster dose. Highlighting the role of vaccination in protecting others could be one way to promote the vaccine and is in line with the “protector schema” [54].

The efficacy of the booster dose, as well as worries about adverse effects, have a significant impact on the reasons for the third dose of the COVID-19 vaccine. Previous research has demonstrated vaccine efficacy, safety and a lack of worry about adverse effects to be associated with increased vaccine acceptance [55]. Despite the government’s efforts to encourage society to acquire the booster vaccine, this study indicated that only 10.6% of participants received the vaccine due to the government’s recommendations. This demonstrates that, in addition to an active government campaign, the message given should include the significance of a booster dose in protecting the individual, the family and the workplace from the threat of COVID-19 [32].

Even if more than half of the participants (53.9%) did not respond, the question regarding the preferred country as the source of the booster dose, the remaining participants agreed that the two most preferred countries are the United States and United Kingdom. This is in line with the perceived superiority of Pfizer and Moderna, which are from the United States, in being the most effective vaccine in terms of protection against hospitalization, admission to the ICU or death [55].

Our study had some limitations that need to be discussed to be able to interpret the results. First, the internet-based survey method in this study may have excluded people who lacked internet access or could not read or write in the country. The active internet user base was approximately 56% of the Indonesian population in 2018 [56]. Young adults are likely more active on social media, and this led to 61% of the respondents in the present study to be younger than 30 years old, which does not reflect on the age distribution of the entire country. Our study can only provide a snapshot of acceptance and/or resistance that might change over time due to its cross-sectional nature. Therefore, a follow-up study could provide trends in acceptance over time. 

## 5. Conclusions

Our study found that the experience of contracting SARS-CoV-2 infection, previously receiving a Sinopharm or AstraZeneca vaccine, and knowledge that vaccines give protection from COVID-19, reduce the length of treatment and protect unvaccinated people could relate to the levels of acceptance of a COVID-19 vaccine booster. Additionally, confidence in their existing immunity, mistrust over the effectiveness of a booster dose, worries regarding the unexpected side effects following a booster dose and concerns about its halal status might inhibit the Indonesian population in accepting the booster dose. 

## Figures and Tables

**Table 1:** Demographic characteristics of the respondents (*n* = 2935).

Characteristic	Number	Percentage
Gender		
Male	942	32.1
Female	1993	67.9
Age (year)		
≤20	391	13.3
21–30	1406	47.9
31–40	758	25.8
41–50	226	7.7
51–60	118	4.0
>60	36	1.2
Marital status		
Single	1556	53.0
Married	1326	45.2
Widow	53	1.8
Educational attainment		
Elementary–Senior High School	625	21.3
Diploma	1973	67.2
Undergraduate/graduated	337	11.5
Religion		
Islam	2376	81.0
Christian (Protestant)	224	7.6
Catholic	162	5.5
Other (Hindu/Buddha/Atheist or Agnostic/Confucian)	173	5.9
Occupation		
Self-employed	36	1.2
Employed for wages	1539	52.4
Homemaker	105	3.6
Student or retired/unable to work/others	1255	42.8
Monthly household income (Indonesian Rupiah)		
<3 million	1638	55.8
3–5 million	456	15.5
5–10 million	530	18.1
>10 million	311	10.6

**Table 2:** Characteristics of the experience, knowledge, perception, perceived severity, perceived benefit and perceived barriers of the respondents (*n* = 2935).

Item	Number	Percentage
Had a family member seriously ill or who died from COVID-19?		
Yes	740	25.2
No	2195	74.8
Had an influenza vaccine in the past 5 years?		
Yes	489	16.7
No	2446	83.3
Have you ever been infected with COVID-19?		
Yes	1387	47.3
No	1548	52.7
Type of COVID-19 vaccine received for the 1st dose		
Sinovac	2498	85.1
AstraZeneca	224	7.6
Moderna	90	3.1
Pfizer	77	2.6
Sinopharm	46	1.6
Type of COVID-19 vaccine received for the 2nd dose		
Sinovac	2368	80.7
AstraZeneca	234	8.0
Moderna	165	5.6
Pfizer	113	3.9
Sinopharm	55	1.9
Have you ever been infected with COVID-19 after getting vaccinated?		
Yes	849	28.9
No	1634	55.7
Do not know	452	15.4
A booster dose can provide better immune than a second dose		
Yes	2389	81.4
No	57	1.9
Not sure	489	16.7
A booster dose can stimulate antibody production to fight COVID-19 infection		
Yes	2546	86.7
No	42	1.4
Not sure	347	11.8
A booster dose can lower hospitalization rate if infected by COVID-19		
Yes	2481	84.5
No	80	2.7
Not sure	374	12.7
A booster dose can protect the unvaccinated people		
Yes	2053	69.9
No	295	10.1
Not sure	587	20.0
The COVID-19 pandemic has greatly affected my source of income		
Agree or strongly agree	2161	73.6
Neither agree nor disagree	663	22.6
Disagree or strongly disagree	111	3.8
The COVID-19 pandemic has greatly affected my social life		
Agree or strongly agree	2591	88.3
Neither agree nor disagree	301	10.3
Disagree or strongly disagree	43	1.5
My decision to be vaccinated with a booster dose was greatly influenced by the workplace during the pandemic		
Agree or strongly agree	2032	69.2
Neither agree nor disagree	632	21.5
Disagree or strongly disagree	271	9.2
The booster dose is important to protect the public from COVID-19		
Yes	2599	88.6
No	77	2.6
Do not know	259	8.8
Pharmaceutical companies have developed a safe and effective booster dose COVID-19 vaccine		
Yes	2214	75.4
No	51	1.7
Do not know	670	22.8
The government provides free booster vaccines for everyone		
Yes	2786	94.9
No	33	1.1
Do not know	116	4.0
I believe that natural immunity is sufficient and I do not need to be vaccinated		
Agree or strongly agree	453	15.4
Neither agree nor disagree	558	19.0
Disagree or strongly disagree	1924	65.6
COVID-19 infection is harmless, so I do not have to be vaccinated		
Agree or strongly agree	274	9.3
Neither agree nor disagree	302	10.3
Disagree or strongly disagree	2359	80.4
My decision to be vaccinated with a booster dose was greatly influenced by the workplace during the pandemic		
Agree or strongly agree	1689	57.5
Neither agree nor disagree	761	25.9
Disagree or strongly disagree	485	16.5
My decision to be vaccinated with a booster dose is strongly influenced by someone or family who lives with me at home		
Agree or strongly agree	1699	57.9
Neither agree nor disagree	694	23.6
Disagree or strongly disagree	542	18.5
I am not sure vaccination is effective against COVID-19		
Agree or strongly agree	482	16.4
Neither agree nor disagree	581	19.8
Disagree or strongly disagree	1872	63.8
I am worried about any adverse side effects or allergic reactions when vaccinated with booster doses		
Agree or strongly agree	1675	57.1
Neither agree nor disagree	887	30.2
Disagree or strongly disagree	373	12.7
I believe a booster dose of COVID-19 vaccine is very important		
Agree or strongly agree	2250	76.7
Neither agree nor disagree	608	20.7
Disagree or strongly disagree	77	2.6
I believe optional vaccines and boosters, as a condition of travel, are necessary and useful		
Agree or strongly agree	2166	73.8
Neither agree nor disagree	525	17.9
Disagree or strongly disagree	244	8.3
A booster dose is useful for protecting people from COVID-19		
Agree or strongly agree	2390	81.4
Neither agree nor disagree	472	16.1
Disagree or strongly disagree	73	2.5
A booster dose is safe		
Agree or strongly agree	2248	76.6
Neither agree nor disagree	627	21.4
Disagree or strongly disagree	60	2.0
Complications may arise after receiving the booster dose		
Agree or strongly agree	424	14.4
Neither agree nor disagree	1170	39.9
Disagree or strongly disagree	1341	45.7
I am worried about the unexpected side effect of booster dose in the future		
Agree or strongly agree	1189	40.5
Neither agree nor disagree	1057	36.0
Disagree or strongly disagree	689	23.5
I believe the booster dose has good effectiveness		
Agree or strongly agree	2242	76.4
Neither agree nor disagree	641	21.8
Disagree or strongly disagree	52	1.8
I believe the booster dose will be useful in protecting me from COVID-19 infection		
Agree or strongly agree	2318	79.0
Neither agree nor disagree	563	19.2
Disagree or strongly disagree	54	1.8
I believe the benefits of the COVID-19 vaccine outweigh the risks		
Agree or strongly agree	2279	77.6
Neither agree nor disagree	601	20.5
Disagree or strongly disagree	55	1.9
I believe if I get vaccinated, the risk of contracting COVID-19 or infecting others will be reduced		
Agree or strongly agree	2401	81.8
Neither agree nor disagree	463	15.8
Disagree or strongly disagree	71	2.4
I am worried about the *halal* status of the new booster dose of COVID-19 vaccine		
Agree or strongly agree	1010	34.4
Neither agree nor disagree	1102	37.5
Disagree or strongly disagree	823	28.0
Getting a booster dose vaccinated takes a lot of time and effort		
Agree or strongly agree	983	33.5
Neither agree nor disagree	1128	38.4
Disagree or strongly disagree	824	28.1

**Table 3:** Acceptance of the COVID-19 vaccine booster dose (*n* = 2935).

Profile of the Vaccine	Number	Percentage
Acceptance of a booster dose of COVID-19 vaccine if it were provided freely by the government (without stating the efficacy or the safety)		
Yes	2758	93.9
No	117	6.1
Acceptance of a booster dose of COVID-19 vaccine if it was 50% effective, with a 5% chance of side effects such as fever.		
Yes	1976	67.3
No	959	32.7
Acceptance of a booster dose of COVID-19 vaccine if it was 95% effective, with a 20% chance of side effects such as fever.		
Yes	2032	69.2
No	903	30.8
Acceptance of a booster dose of COVID-19 vaccine if it was 75% effective, with a 5% chance of side effects such as fever.		
Yes	2461	83.9
No	474	16.1
Acceptance of a booster dose of COVID-19 vaccine if it was 75% effective, with a 20% chance of side effects such as fever.		
Yes	1477	50.3
No	1458	49.7

**Table 4:** Initial multivariable linear regression model showing the factors associated with acceptance for a booster dose of the COVID-19 vaccine in Indonesia (*n* = 2935).

Item	Number	Percentage	Acceptance Yes	Univariate		Multivariate	
			*n* (%)	OR 95%CI	*p* Value	OR 95%CI	*p* Value
Gender							
Male	942	32.1	880 (93.4)	1			
Female	1993	67.9	1878 (94.2)	1.15 (0.84–1.58)	0.389		
Age							
≤20	391	13.3	380 (97.2)	3.30 (1.72–6.31)	<0.001	3.51 (1.30–9.46)	0.013
21–30	1406	47.9	1327 (94.4)	1.60 (1.14–2.25)	0.007	1.96 (1.10–3.50)	0.022
31–40	758	25.8	692 (91.3)	1		1	
41–50	226	7.7	211 (93.4)	1.34 (0.75–2.40)	0.322	1.76 (0.71–4.35)	0.220
51–60	118	4.0	113 (95.8)	2.16 (0.85–5.47)	0.106	1.59 (0.40–6.31)	0.508
>60	36	1.2	35 (97.2)	3.34 (0.45–24.76)	0.238	1.79 (0.12–25.98)	0.671
Marital status							
Single	1556	53.0	1475 (94.8)	1		1	
Married	1326	45.2	1232 (92.9)	0.72 (0.53–0.98)	0.036	1.21 (0.69–2.13)	0.514
Widow	53	1.8	51 (96.2)	1.40 (0.34–5.85)	0.645	1.55 (0.17–14.56)	0.702
Educational attainment							
Elementary–Senior High School	625	21.3	596 (95.4)	1			
Diploma	1973	67.2	1847 (93.6)	0.71 (0.47–1.08)	0.110		
Undergraduate/graduated	337	11.5	315 (93.5)	0.70 (0.39–1.23)	0.215		
Religion							
Islam	2376	81.0	2208 (92.9)	1		1	
Christian (Protestant)	224	7.6	219 (97.8)	3.33 (1.36–8.20)	0.009	2.67 (0.86–8.31)	0.091
Catholic	162	5.5	158 (97.5)	3.01 (1.10–8.21)	0.032	2.13 (0.57–7.99)	0.262
Other (Hindu/Buddha/Atheist or Agnostic/Confucian)	173	5.9	173 (100.0)	1 × 10^8^ (0.00–NA)	0.995	3 × 10^7^ (0.00–NA)	0.995
Occupation						2.51 (0.43–14.75)	0.309
Self-employed	36	1.2	33 (91.7)	1.83 (0.50–6.74)	0.362	2.57 (1.07–6.18)	0.035
Employed for wages	1539	52.4	1450 (94.2)	2.72 (1.51–4.88)	0.001	1	
Homemaker	105	3.6	90 (85.7)	1		1.89 (0.74–4.82)	0.181
Student or retired/unable to work/others	1255	42.8	1185 (94.4)	12.82 (1.55–5.13)	0.001		
Monthly household income (Indonesian Rupiah)							
<3 million	1638	55.8	1536 (93.8)	1			
3–5 million	456	15.5	418 (91.7)	0.73 (0.50–1.08)	0.113		
5–10 million	530	18.1	507 (95.7)	1.46 (0.92–2.33)	0.107		
>10 million	311	10.6	297 (95.5)	1.41 (0.80–2.50)	0.240		
Had a family member seriously ill or who died from COVID-19?							
Yes	740	25.2	706 (95.4)	1.45 (0.99–2.12)	0.059		
No *(R)*	2195	74.8	2052 (93.5)	1			
Had an influenza vaccine in the past 5 years?							
Yes	489	16.7	460 (94.1)	1.02 (0.68–1.54)	0.919		
No *(R)*	2446	83.3	2298 (93.9)	1			
Have ever been infected with COVID-19?							
Yes	1387	47.3	1322 (95.3)	1.59 (1.16–2.17)	0.004	1.25 (0.74–2.10)	0.403
No *(R)*	1548	52.7	1436 (92.8)	1		1	
Type of COVID-19 vaccine you received for the 1st dose							
Sinovac *(R)*	2498	85.1	2362 (94.6)	1		1	
AstraZeneca	224	7.6	209 (93.3)	0.81 (0.46–1.39)	0.434	0.30 (0.08–1.05)	0.059
Moderna	90	3.1	83 (92.2)	0.68 (0.31–1.51)	0.344	2.58 (0.64–10.40)	0.183
Pfizer	77	2.6	62 (80.5)	0.24 (0.13–0.43)	<0.001	0.43 (0.09–2.10)	0.296
Sinopharm	46	1.6	42 (91.3)	0.61 (0.21–1.71)	0.343	0.08 (0.01–0.68)	0.020
Type of COVID-19 vaccine you received for the 2nd dose							
Sinovac *(R)*	2368	80.7	2240 (94.6)	1		1	
AstraZeneca	234	8.0	226 (96.6)	1.61 (0.78–3.34)	0.197	7.72 (1.84–32.42)	0.005
Moderna	165	5.6	146 (88.5)	0.44 (0.26–0.73)	0.002	0.40 (0.15–1.06)	0.067
Pfizer	113	3.9	95 (84.1)	0.30 (0.18–0.52)	<0.001	1.44 (0.35–5.95)	0.617
Sinopharm	55	1.9	51 (92.7)	0.73 (0.26–2.05)	0.548	7.38 (0.81–67.30)	0.076
Have you ever been infected with COVID-19 after getting vaccinated?							
Yes *(R)*	849	28.9	821 (96.7)	1		1	
No	1634	55.7	1560 (95.5)	0.72 (0.46–1.12)	0.144	0.82 (0.41–1.63)	0.563
Do not know	452	15.4	377 (83.4)	0.17 (0.11–0.27)	<0.001	0.42 (0.21–0.86)	0.018
A booster dose can provide better immune than a second dose							
Yes *(R)*	2389	81.4	2329 (97.5)	1		1	
No	57	1.9	37 (64.9)	0.05 (0.03–0.09)	<0.001	0.91 (0.28–2.97)	0.877
Not sure	489	16.7	392 (80.2)	0.10 (0.07–0.15)	<0.001	0.74 (0.38–1.47)	0.393
A booster dose can stimulate antibody production to fight COVID-19							
Yes *(R)*	2546	86.7	2476 (97.3)	1		1	
No	42	1.4	24 (57.1)	0.04 (0.02–0.07)	<0.001	0.34 (0.09–1.27)	0.109
Not sure	347	11.8	258 (74.4)	0.08 (0.06–0.12)	<0.001	0.77 (0.37–1.58)	0.469
A booster dose can lower hospitalization rate if infected by COVID-19							
Yes *(R)*	2481	84.5	2415 (97.3)	1		1	
No	80	2.7	64 (80.0)	0.11 (0.06–0.20)	<0.001	0.88 (0.29–2.62)	0.816
Not sure	374	12.7	279 (74.6)	0.08 (0.06–0.11)	<0.001	0.69 (0.35–1.34)	0.268
A booster dose can protect the unvaccinated people							
Yes *(R)*	2053	69.9	1994 (97.1)	1		1	
No	295	10.1	267 (90.5)	0.28 (0.18–0.45)	<0.001	0.81 (0.39–1.69)	0.579
Not sure	587	20.0	497 (84.7)	0.16 (0.12–0.23)	<0.001	1.33 (0.72–2.46)	0.371
The COVID-19 pandemic has greatly affected my source of income							
Agree or strongly agree	2161	73.6	2042 (94.5)	2.08 (1.11–3.89)	0.022	1.21 (0.41–3.57)	0.731
Neither agree nor disagree	663	22.6	617 (93.1)	1.63 (0.83–3.18)	0.155	1.28 (0.42–3.92)	0.665
Disagree or strongly disagree *(R)*	111	3.8	99 (89.2)	1		1	
The COVID-19 pandemic has greatly affected my social life							
Agree or strongly agree	2591	88.3	2454 (94.7)	5.43 (2.62–11.24)	<0.001	2.04 (0.47–8.84)	0.340
Neither agree nor disagree	301	10.3	271 (90.0)	2.74 (1.23–6.10)	0.014	1.12 (0.24–5.16)	0.889
Disagree or strongly disagree *(R)*	43	1.5	33 (76.7)	1		1	
My decision to be vaccinated with a booster dose was greatly influenced by the workplace during the pandemic							
Agree or strongly agree	2032	69.2	1926 (94.8)	1.53 (0.94–2.48)	0.088		
Neither agree nor disagree	632	21.5	582 (92.1)	0.98 (0.58–1.66)	0.934		
Disagree or strongly disagree *(R)*	271	9.2	250 (92.3)	1			
The booster dose is important to protect the public from COVID-19							
Yes	2599	88.6	2531 (97.4)	38.20 (23.00–63.46)	<0.001	3.17 (1.34–7.50)	0.009
No *(R)*	77	2.6	38 (49.4)	1		1	
Do not know	259	8.8	189 (73.0)	2.77 (1.64–4.68)	<0.001	3.02 (1.28–7.16)	0.012
Pharmaceutical companies have developed a safe and effective booster dose of COVID-19 vaccine							
Yes	2214	75.4	2166 (97.8)	26.79 (14.19–50.59)	<0.001	0.79 (0.25–2.54)	0.691
No *(R)*	51	1.7	32 (62.7)	1		1	
Do not know	670	22.8	560 (83.6)	3.02 (1.65–5.53)	<0.001	0.55 (0.18–1.66)	0.285
I believe that natural immunity is sufficient and I do not need to be vaccinated							
Agree or strongly agree	453	15.4	367 (81.0)	0.08 (0.05–0.12)	<0.001	0.43 (0.23–0.83)	0.011
Neither agree nor disagree	558	19.0	502 (90.0)	0.17 (0.11–0.26)	<0.001	0.95 (0.51–1.77)	0.872
Disagree or strongly disagree *(R)*	1924	65.6	1889 (98.2)	1		1	
COVID-19 infection is harmless, so I do not have to be vaccinated							
Agree or strongly agree	274	9.3	225 (82.1)	0.13 (0.09–0.19)	<0.001	0.62 (0.30–1.28)	0.195
Neither agree nor disagree	302	10.3	239 (79.1)	0.11 (0.07–0.16)	<0.001	0.65 (0.36–1.15)	0.137
Disagree or strongly disagree *(R)*	2359	80.4	2294 (97.2)	1		1	
My decision to be vaccinated with a booster dose was greatly influenced by the workplace during the pandemic							
Agree or strongly agree	1689	57.5	1586 (93.9)	0.80 (0.51–1.27)	0.342		
Neither agree nor disagree	761	25.9	711 (93.4)	0.74 (0.45–1.22)	0.239		
Disagree or strongly disagree *(R)*	485	16.5	461 (95.1)	1			
My decision to be vaccinated with a booster dose is strongly influenced by someone or family who lives with me at home							
Agree or strongly agree	1699	57.9	1637 (96.4)	2.39 (1.61–3.56)	<0.001	1.80 (0.99–3.28)	0.055
Neither agree nor disagree	694	23.6	624 (89.9)	0.81 (0.54–1.20)	0.285	1.51 (0.82–2.77)	0.188
Disagree or strongly disagree *(R)*	542	18.5	497 (91.7)	1		1	
I’m not sure vaccination is effective against COVID-19							
Agree or strongly agree	482	16.4	400 (83.0)	0.08 (0.05–0.12)	<0.001	0.42 (0.21–0.83)	0.012
Neither agree nor disagree	581	19.8	515 (88.6)	0.12 (0.08–0.19)	<0.001	0.46 (0.24–0.87)	0.017
Disagree or strongly disagree *(R)*	1872	63.8	1843 (98.5)	1		1	
I am worried about any adverse side effects or allergic reactions when vaccinated with booster doses							
Agree or strongly agree	1675	57.1	1534 (91.6)	0.30 (0.16–0.58)	<0.001	1.19 (0.40–3.57)	0.754
Neither agree nor disagree	887	30.2	861 (97.1)	0.91 (0.44–1.91)	0.808	1.63 (0.52–5.09)	0.401
Disagree or strongly disagree *(R)*	373	12.7	363 (97.3)	1		1	
I believe a booster dose of COVID-19 vaccine is very important							
Agree or strongly agree	2250	76.7	2215 (98.4)	93.91 (53.39–165.18)	<0.001	5.54 (1.90–16.15)	0.002
Neither agree nor disagree	608	20.7	512 (84.2)	7.91 (4.78–13.11)	<0.001	2.46 (0.97–6.20)	0.057
Disagree or strongly disagree *(R)*	77	2.6	31 (40.3)	1		1	
I believe optional vaccines and boosters as a condition of travel, are necessary and useful							
Agree or strongly agree	2166	73.8	2102 (97.0)	10.47 (7.13–15.38)	<0.001	0.76 (0.39–1.48)	0.413
Neither agree nor disagree	525	17.9	471 (89.7)	2.78 (1.85–4.18)	<0.001	1.14 (0.61–2.15)	0.680
Disagree or strongly disagree *(R)*	244	8.3	185 (75.8)	1		1	
A booster vaccines are useful for protecting people from COVID-19							
Agree or strongly agree	2390	81.4	2342 (98.0)	55.97 (32.57–96.18)	<0.001	0.78 (0.24–2.59)	0.685
Neither agree nor disagree	472	16.1	382 (80.9)	4.87 (2.91–8.14)	<0.001	0.86 (0.29–2.57)	0.791
Disagree or strongly disagree *(R)*	73	2.5	34 (46.6)	1		1	
A booster vaccine is safe							
Agree or strongly agree	2248	76.6	2207 (98.2)	57.54 (31.80–104.13)	<0.001	2.74 (0.97–7.76)	0.057
Neither agree nor disagree	627	21.4	522 (83.3)	5.31 (3.07–9.19)	<0.001	1.79 (0.69–4.61)	0.231
Disagree or strongly disagree *(R)*	60	2.0	29 (48.3)	1		1	
Serious complications will arise after getting the COVID-19 booster vaccine							
Agree or strongly agree	424	14.4	379 (89.4)	0.19 (0.12–0.31)	<0.001	1.32 (0.61–2.84)	0.479
Neither agree nor disagree	1170	39.9	1068 (91.3)	0.24 (0.16–0.36)	<0.001	1.07 (0.57–2.04)	0.830
Disagree or strongly disagree *(R)*	1341	45.7	1311 (97.8)	1		1	
I am worried about the unexpected effect of the booster dose in the future							
Agree or strongly agree	1189	40.5	1071 (90.1)	0.12 (0.06–0.24)	<0.001	0.67 (0.23–1.93)	0.456
Neither agree nor disagree	1057	36.0	1007 (95.3)	0.27 (0.13–0.55)	<0.001	0.61 (0.21–1.75)	0.360
Disagree or strongly disagree *(R)*	689	23.5	680 (98.7)	1		1	
I believe the booster dose will have good effectiveness							
Agree or strongly agree	2242	76.4	2205 (98.3)	69.53 (36.86–131.16)	<0.001	0.39 (0.06–2.40)	0.310
Neither agree nor disagree	641	21.8	529 (82.5)	5.51 (3.08–9.86)	<0.001	0.31 (0.06–1.60)	0.162
Disagree or strongly disagree *(R)*	52	1.8	24 (46.2)	1		1	
I believe the COVID-19 booster dose vaccine will be useful in protecting me from COVID-19 infection							
Agree or strongly agree	2318	79.0	2272 (98.0)	98.78 (52.26–186.71)	<0.001	3.29 (0.53–20.45)	0.201
Neither agree nor disagree	563	19.2	468 (83.1)	9.85 (5.37–18.08)	<0.001	4.52 (0.83–24.66)	0.081
Disagree or strongly disagree *(R)*	54	1.8	18 (33.3)	1		1	
I believe the benefits of COVID-19 vaccine outweigh the risks							
Agree or strongly agree	2279	77.6	2239 (98.2)	90.63 (48.39–169.72)	<0.001	2.45 (0.67–8.89)	0.173
Neither agree nor disagree	601	20.5	498 (82.9)	7.83 (4.37–14.04)	<0.001	1.20 (0.36–4.00)	0.766
Disagree or strongly disagree *(R)*	55	1.9	21 (38.2)	1		1	
I believe that if I get vaccinated, the risk of contracting COVID-19 or infecting others will be reduced							
Agree or strongly agree	2401	81.8	2348 (97.8)	34.33 (19.96–59.05)	<0.001	0.87 (0.26–2.94)	0.820
Neither agree nor disagree	463	15.8	370 (79.9)	3.08 (1.83–5.19)	<0.001	0.87 (0.27–2.75)	0.809
Disagree or strongly disagree *(R)*	71	2.4	40 (56.3)	1		1	
I am worried about the *halal* status of the new booster dose of the COVID-19 vaccine							
Agree or strongly agree	1010	34.4	910 (90.1)	0.20 (0.12–0.34)	<0.001	1.56 (0.71–3.45)	0.270
Neither agree nor disagree	1102	37.5	1043 (94.6)	0.40 (0.23–0.68)	0.001	1.75 (0.79–3.88)	0.171
Disagree or strongly disagree *(R)*	823	28.0	805 (97.8)	1		1	
Having a booster dose takes a lot of time and effort							
Agree or strongly agree	983	33.5	922 (93.8)	0.57 (0.37–0.89)	0.014	0.98 (0.50–1.93)	0.958
Neither agree nor disagree	1128	38.4	1042 (92.4)	0.46 (0.30–0.70)	<0.001	1.12 (0.57–2.19)	0.739
Disagree or strongly disagree *(R)*	824	28.1	794 (96.4)	1		1	

## Data Availability

Underlying data of the present study are available from the corresponding author with acceptable reasons.
